# Extracellular vesicle-packaged miR-4253 secreted by cancer-associated fibroblasts facilitates cell proliferation in gastric cancer by inducing macrophage M2 polarization

**DOI:** 10.1080/15384047.2024.2424490

**Published:** 2024-11-06

**Authors:** Xinxing Duan, Xiong Yu, Jin Gan

**Affiliations:** General Surgery Center, Jiujiang City Key Laboratory of Cell Therapy, Jiujiang, China

**Keywords:** Gastric cancer, extracellular vesicle, macrophage polarization, cancer-associated fibroblast, miR-4253, IL6R

## Abstract

Cancer-associated fibroblasts (CAFs) can interact with macrophages in the tumor microenvironment by secreting extracellular vesicles (EVs), thereby affecting tumor progression. However, the mechanisms of CAF-secreted EVs in gastric cancer (GC) remain not well understood. Here, we investigated the effect of CAF-EVs on macrophage polarization in GC and the underlying mechanisms. Macrophage polarization was evaluated using flow cytometry and quantitative real-time polymerase chain reaction. GC cell proliferation was determined using cell counting kit-8, EdU, and colony formation assays. The molecular mechanism was explored using microarray analysis, dual-luciferase reporter assay, and RNA pull-down analysis. The results showed that CAFs secreted EVs that inhibit macrophage M1 polarization and promote M2 polarization. Moreover, miR-4253 expression was increased in CAF-EVs, and inhibition of miR-4253 reversed the macrophage polarization induced by EVs. IL6R was identified as the target of miR-4253. Additionally, macrophages treated with EVs that encapsulated miR-4253 promote GC cell proliferation. In conclusion, CAF-secreted EVs packaging miR-4253 facilitate macrophage polarization from M1 to M2 phenotype by targeting IL6R, thereby accelerating GC cell proliferation. The findings suggest that EV-encapsulated miR-4253 may be a promising therapeutic target of GC.

## Graphical abstract

**Figure:**
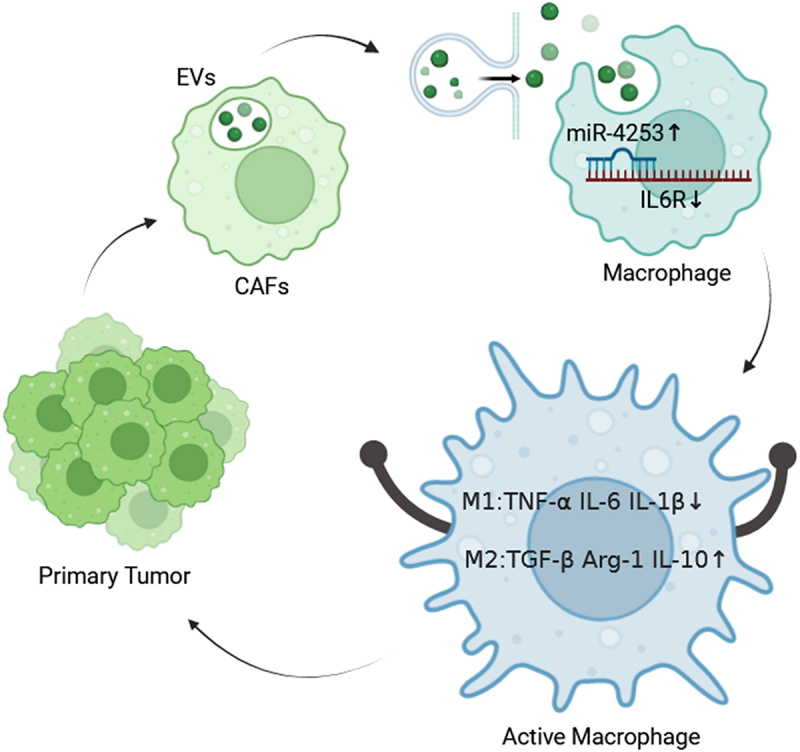

## Introduction

Gastric cancer (GC) is a global burden with over one million new cases and 768,793 new deaths in 2020.^1^ Despite a decrease in the overall GC burden, it still ranks as the third leading type of malignancy with the third leading cause of cancer death in China.^2^ Due to the early asymptomatic and low screening rates, most patients are diagnosed at advanced stages. At present, perioperative chemotherapy is the standard treatment for early localized GC, while immunotherapy and targeted therapies have been developed for advanced stages.^3^ However, difficult-to-control metastases and recurrence continue to be the major causes of high mortality rates.^4,5^ Therefore, elucidating the pathological physiology of GC is necessary.

Various types of cells in the tumor microenvironment (TME) play a crucial role in regulating tumor survival, growth, and metastasis, including cancer-associated fibroblasts (CAFs) and tumor-associated macrophages (TAMs).^6,7^ Evidence has been confirmed that CAFs interact with TAMs by secreting several molecules, such as cytokines, chemokines, extracellular vesicles (EVs), and growth factors, creating an immunosuppressive TME that facilitates cancer cell immune escape.^8^ EVs containing nucleic acids, lipids, and proteins are involved in cell-cell communication.^9^ CAFs release EVs to regulate the processes of tumor cells or other cells in TME, ultimately affecting tumor cell proliferation, metastasis, metabolic reprogramming, and therapeutic resistance.^10^ Macrophage polarization can be mediated by EVs. TAMs typically polarize into M1 and M2 phenotypes, which serve pro-inflammatory/anti-tumor and anti-inflammatory/pro-tumor functions, respectively.^11^ Hence, clarifying the regulatory mechanisms governing communication between CAFs and TAMs is essential for understanding GC progression.

MicroRNAs (miRNAs) are small non-coding RNAs that can be encapsulated within EVs and transported to neighboring cells. They regulate the expression of target mRNAs by binding to their 3′untranslated region (3’UTR), thereby influencing genomic stability.^12^ MiRNAs are commonly aberrantly expressed in cancers and play an important role in tumor growth, immunity, resistance, metastasis, and death.^13,14^ Importantly, miRNAs are implicated in macrophage polarization via the capability to drive M1 and M2 polarization.^15^ They show promise as diagnostic and prognostic biomarkers and as potential therapeutic targets. However, the understanding of miRNA functions and regulatory networks is not comprehensive, necessitating further research.

In this study, we investigated the effect of CAF-EVs on macrophage polarization and CG cell proliferation using in vitro experiments. Moreover, the molecular mechanisms of EV function were investigated. This study may aid in enhancing our comprehension of GC progression.

## Materials and methods

### Cell culture and treatment

GC-associated fibroblasts (CAFs) were purchased from SAIOS (Wuhan, China) and were cultured in fibroblast culture media (SAIOS) at 37°C with 5% CO_2_ atmosphere. To identify CAFs, cell morphology was observed under a light microscope. The markers of CAFs including αSMA and FAP were measured using immunofluorescence.

Human monocyte cell line THP-1 was acquired from Procell (Wuhan, China). The cells were cultured at RPMI-1640 (Gibco) supplemented with 10% fetal bovine serum (FBS; Gibco), 0.05 mm β-mercaptoethanol (Sigma-Aldrich), and 1% penicillin-streptomycin solution (Procell) at 37°C with 5% CO_2_ atmosphere. To induce macrophage, THP-1 cells were exposed to 100 ng/mL phorbol 12-myristate 13-acetate (PMA; Sigma-Aldrich) for 48 h. To generate a cell co-culture system, CAFs were co-cultured with macrophages for 48 h. GW4869 (10 mm; MedChemExpress, Monmouth Junction, NJ, US) was added to the culture medium to inhibit EV biogenesis or release.

To induce M1 macrophages, PMA-differentiated THP-1 cells were treated with 100 ng/mL LPS (Sigma-Aldrich) and 20 ng/mL IFN-γ (Sigma-Aldrich) for 48 h. To induce M2 macrophages, PMA-differentiated THP-1 cells were treated with 20 ng/mL IL-4 (Sigma-Aldrich) and 20 ng/mL IL-13 (Sigma-Aldrich) for 48 h.^16^

GC cell line (AGS) was acquired from ATCC and maintained at RPMI-1640 containing 10% FBS and 1% penicillin-streptomycin at 37°C with 5% CO_2_.

### Immunofluorescence

CAF slices were fixed with 4% paraformaldehyde for 20 min and permeated with 0.5% Triton X-100 for 20 min. Next, the slices were blocked in normal goat serum for 30 min. Primary antibodies including anti-αSMA and anti-FAP were incubated with cell slices overnight at 4°C, and then labeled with fluorescent-dye conjugated secondary antibodies at 37°C for 1 h. 4′,6-diamidino-2-phenylindole (DAPI) was incubated with the cells to stain nuclei. Images were captured under a fluorescent microscope.

### EV isolation

EVs were isolated from CAFs using ultracentrifugation as previously described with minor changes.^17^ The culture medium of CAFs was collected by centrifuging at 300 *g* for 10 min, and then cell debris was removed by centrifuging at 3,000 *g* for 20 min. The medium was centrifuged at 10,000 *g* for 30 min and 120,000 *g* for 70 min. The EVs were collected and suspended in PBS for use. EV morphology was observed using a transmission electron microscope (TEM), particle size was measured using nanoparticle tracking analysis (NTA), and the levels of surface markers were examined using immunoblotting.

### PKH67 staining

EV tracing was evaluated using the PKH67 fluorescent dye (BestBio, Shanghai, China). Briefly, EVs were incubated with PKH67 solution at 37°C for 30 min in the dark. Then, PKH67-labeled EVs were incubated with macrophages at 37°C for 48 h. 4′,6-diamidino-2-phenylindole (DAPI; 1 μg/mL; BestBio) was incubated with cells for 15 min to stain cell nuclei. After fixing with 4% paraformaldehyde, EV internalization was viewed using a laser scanning confocal microscope.

### Cell transfection

Short hairpin RNA (shRNA) targeting Dicer (sh-Dicer), shRNA targeting IL6R (sh-IL6R), and their negative control (sh-NC) were synthesized by GenePharma (Shanghai, China). miR-4253 inhibitor, mimic, and their negative control (nc inhibitor and nc mimic) were acquired from RiboBio (Guangzhou, China). sh-NC, sh-Dicer, nc inhibitor, and miR-4253 inhibitor were transfected into CAFs, as well as nc inhibitor, miR-4253 inhibitor, sh-NC and sh-IL6R were transfected into THP-1 cells using Lipofectamine 2000 (Invitrogen, Carlsbad, CA, USA) for 48 h in line with manufacturer’s protocols.

### Quantitative real-time polymerase chain reaction (qPCR)

Total RNA was isolated from cells using TRIzol reagent (Invitrogen). To detect mRNA expression, reverse transcription (RT) was conducted using the MightyScript first strand cDNA synthesis master mix (Sangon, Shanghai, China). The synthesized cDNA was used for qPCR using the SGExcel FastSYBR mixture (Sangon). To measure miRNA expression, RT and qPCR were carried out using the miDETECT A Track miRNA qRT-PCR starter kit (RiboBio) according to the manufacturer’s protocol. The relative expression was calculated using the 2^−ΔΔCt^ method. GAPDH and U6 were the internal controls of mRNAs and miRNAs, respectively.

### Flow cytometry

Macrophages were washed with PBS and incubated with Fc block (anti-mouse CD16/CD32; BD Biosciences, San Jose, CA, USA) for 15 min. Subsequently, the cells were incubated with FITC-conjugated anti-human CD68 and PE-conjugated anti-human CD86 or APC-conjugated anti-human CD206 (BioLegend, San Diego, CA, USA) for 30 min away from light. Cells were resuspended in PBS and analyzed using a flow cytometer.

### Microarray analysis

The GSE224056 dataset was derived from the Gene Expression Omnibus database and was analyzed using GEO2R. The differentially expressed miRNAs were defined as *p* < .05 and |log(fold change)| > 1.

### Dual-luciferase reporter analysis

The targets of miR-4253 were predicted using the TargetScan database. To verify the targeting relation, wild-type IL6R sequences containing the potential binding sites were cloned into the pmiR-GLO vector (Promega, Madison, WI, USA), named IL6R-wt. Additionally, IL-6 R with mutated potential binds site sequences were also inserted into the pmiR-GLO vector, named IL6R-mut. Macrophages were co-transfected with IL6R-wt or IL6R-mut and nc mimic/nc inhibitor or miR-4253 mimic/inhibitor using Lipofectamine 2000. After 48 h, the firefly and *Renilla* luciferase activities were measured using the dual-luciferase reporter assay system (Promega).

### RNA pull-down assay

Biotin-labeled miR-4253 and its negative control (biotin-nc) were incubated with M‐280 streptavidin magnetic beads (Invitrogen) for 2 h to form the probe-beads mixture. Macrophages were lysed and incubated with the mixture at 4°C for a night. After washing, the complexes were eluted. RNA was isolated, and IL6R expression was measured using qPCR.

### Cell counting kit-8 (CCK-8)

AGS cells in the different groups were seeded into 96-well plates (2 × 10^3^ cells) for 48 h. CCK-8 (10 μL; Dojindo, Kumamoto, Japan) was incubated with the cells to evaluate cell viability for 4 h. The absorbance was measured at 450 nm using a microplate reader.

### EdU assay

A Cell-Light^TM^ EdU Apollo in vitro kit (RibiBio) was used to evaluate cell proliferation. AGS cells were seeded into 96-well plates and labeled with 100 μL 50 μM EdU solution for 2 h. The cells were immobilized with 4% paraformaldehyde and permeabilized using 0.5% TritonX-100 for 30 min and 10 min, respectively. Apollo reaction solution was added to each well to incubate with the cells for 30 min. Cell nuclei were stained with DAPI. Cells were observed using a laser scanning confocal microscope.

### Colony formation assay

AGS cells in the different groups were seeded into 6-well plates for 2 weeks at 37°C. The colonies were observed under a light microscope.

### Statistical analysis

All experiments were performed in triplicate. Data were analyzed using the GraphPad Prism 8.0 software, and the results were expressed as mean ± standard deviation. T-test and one-way ANOVA were used to compare the differences. *p* < .05 was considered statistically significant.

## Results

### CAFs inhibit macrophage M1 polarization and promote M2 polarization

**Figure 1.**
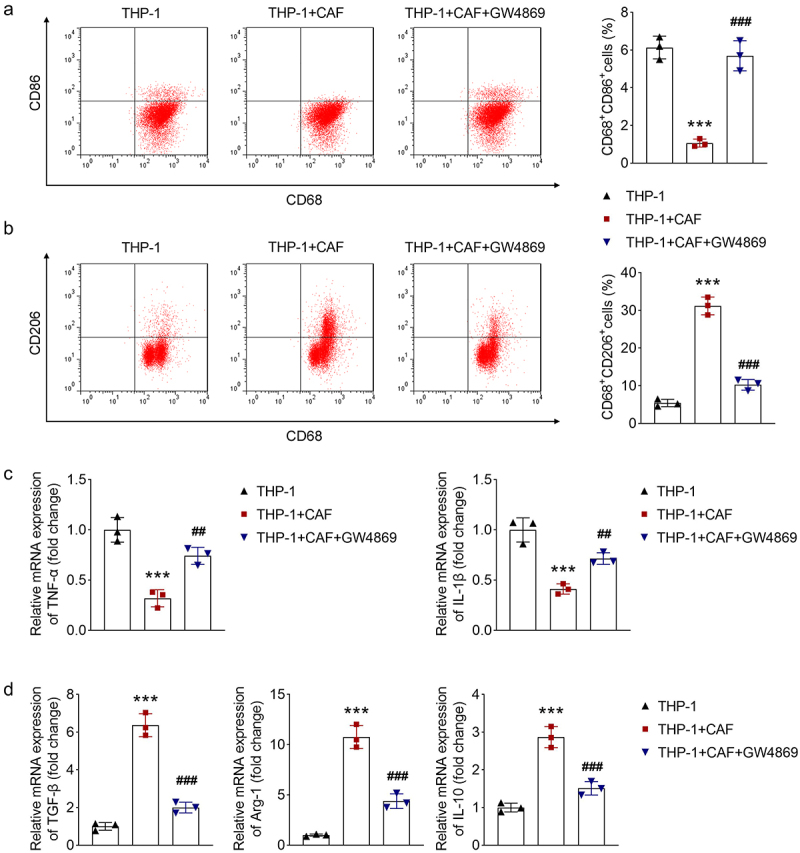
CAFs inhibit macrophage M1 polarization and promote M2 polarization. Macrophages were co-cultured with CAFs, and GW4869 was added to the co-culture system. Macrophage polarization was analyzed. (a) CD68^+^CD86^+^ and (b) CD68^+^CD206^+^ cells were measured using flow cytometry. (c) M1 markers including tnf-α and IL-1β, as well as (d) M2 markers including tgf-β, Arg-1, and IL-10 were measured using qPCR. ***p < .001 and **p < .01 vs. The THP-1 group. ###*P* < .001 and ##*P* < .01 vs. The THP-1 + CAF group. CAF. cancer-associated fibroblast.

To explore the effect of CAFs on macrophage polarization, we first identified the CAFs. We observed that CAFs exhibited spindle-shaped morphology (Supplementary Figure. S1A). FAP and αSMA are CAF markers.^18^ We measured their levels using immunofluorescence. We found that the cells could express αSMA and FAP (Supplementary Figure. S1B). Thus, the cells were confirmed as CAFs. GW4869 was considered to inhibit EV biogenesis and release. To confirm this, we isolated EVs from non-treated CAFs and GW4869-treated CAFs and treated them with THP-1 cells. We found that CD63 and TSG101 levels after treatment with EVs from non-treated CAFs were higher than treatment with EVs from GW4869-treated CAFs (Supplementary Figure. S2), suggesting that GW4869 treatment can reduce EVs. To explore whether EVs are involved in communication between CAFs and macrophages. THP-1 cells were stimulated by PMA to induce M0 macrophages, followed by co-cultured with CAFs, and GW4869 was added to the co-culture system. We determined macrophage polarization using flow cytometry and qPCR. Flow cytometry results indicated that CAFs reduced CD86 levels and increased CD206 levels, whereas GW4869 reversed the effect caused by CAFs (Figure 1a,b). The results of qPCR showed that the expression of TNF-α and IL-1β was decreased in the co-culture system, whereas GW4869 reversed this decrease (Figure 1c). Inversely, the expression of TGF-β, Arg-1, and IL-10 was elevated in the co-culture system, which was rescued by GW4869 treatment (Figure 1d). Moreover, we found that GW4869 alone could not affect macrophage polarization (Supplementary Figure. S3A and B). Taken together, CAFs inhibit M1 polarization and promote M2 polarization of macrophages, at least in part through the secretion of EVs.

### CAF-EVs can be ingested by macrophages

**Figure 2.**
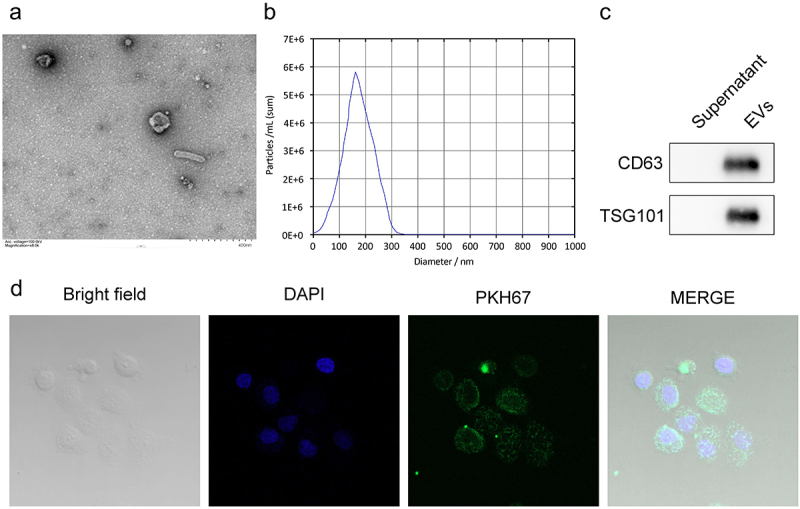
CAF-EVs can be ingested by macrophages. (a) The morphology of EVs isolated from CAFs was photographed using TEM. (b) The size of EVs was measured using NTA. (c) Immunoblotting was performed to measure EV markers including CD63 and TSG101. (d) PKH67-labeled EVs were incubated with macrophages, and the fluorescence signals were observed. EV, extracellular vesicle.

As mentioned above, CAFs exert their functions through the EVs, therefore, we isolated EVs from CAFs. EVs were photographed using TEM and the results showed that their morphology was consistent with that of EVs (Figure 2a). NTA was used to measure the size of the particles, and the results showed that their diameter was consistent with that of EVs (Fig, 2B). Moreover, the markers of EVs were also measured. Immunoblotting results indicated that CD63 and TSG101 were expressed in EVs (Figure 2c). The results demonstrate that EVs were isolated from CAFs. Next, we analyzed whether macrophages could uptake CAF-EVs by co-cultivation of macrophages and PKH67-stained EVs. We found that macrophages could uptake EVs (Figure 2d), suggesting that CAF-secreted EVs can be internalized by macrophages to regulate macrophage functions.

### CAF-EVs regulate macrophage polarization by regulating Dicer

**Figure 3.**
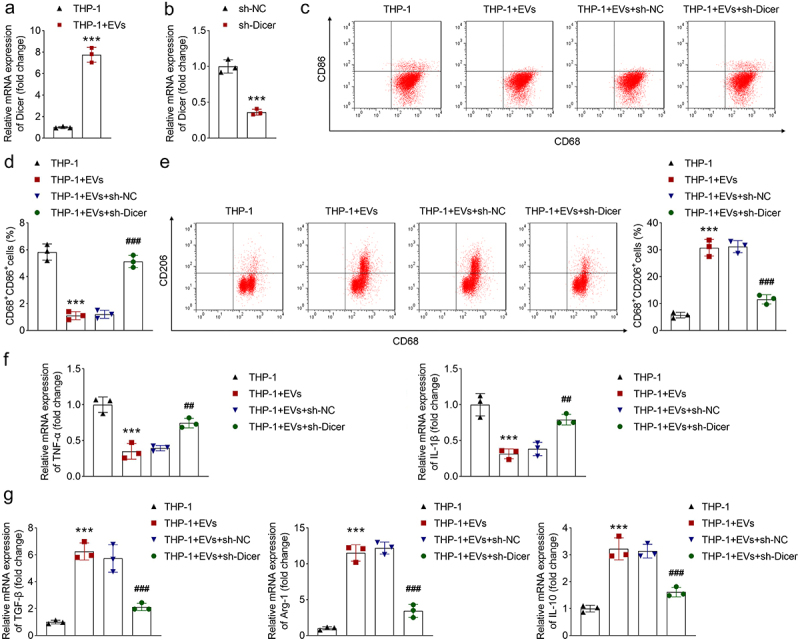
CAF-EVs regulate macrophage polarization by regulating Dicer. (a) The expression of Dicer was measured using qPCR in macrophages treated with EVs. (b) qPCR was performed to measure Dicer expression after sh-nc and sh-Dicer transfection. Macrophages were transfected with sh-nc or sh-Dicer, and treated with EVs or not, (c-e) CD86 and CD206 levels were measured using flow cytometry; the expression of (f) tnf-α, IL-1β, (g) tgf-β, Arg-1, and IL-10 was determined using qPCR. ***p < .001 and **p < .01 vs. The sh-nc or THP-1 group. ###*P* < .001 and ##*P* < .01 vs. The THP-1 + EVs + sh-nc group. EV, extracellular vesicle; sh, short hairpin RNA; nc, negative control.

In THP-1 cells, after EV treatment, the expression of Dicer was increased (Figure 3a), suggesting that EVs contain Dicer. Human Dicer was used to cleave precursor miRNAs to produce mature miRNAs.^19^ Then, mature miRNAs are secreted from EVs under lysosomal digestion.^20^ To explore whether CAF-released EVs carry miRNAs, we knocked down Dicer in CAFs. qPCR results showed that sh-Dicer transfection downregulated Dicer expression (Figure 3b). Then, we collected EVs from transfected CAFs and added them to the co-culture medium of macrophages. CD86 levels were reduced, and CD206 levels were elevated after EV treatment, which was rescued by Dicer silenced (Figure 3c-e). EVs downregulated TNF-α and IL-1β expression, whereas Dicer knockdown counteracted this decrease (Figure 3f). Additionally, EVs upregulated TGF-β, Arg-1, and IL-10 expression, which was abrogated by Dicer knockdown (Figure 3g). The data indicate that EVs suppresses macrophage M1 polarization and facilitate M2 polarization, while knockdown of Dicer has the opposite function. To further investigate the effect of EVs on macrophage polarization. We treated THP-1 cells with LPS and IFN-γ to induce M1 macrophages, and also treated them with IL-4 and IL-13 to induce M2 polarization. EVs were used to treat M1 or M2 cells, and flow cytometry was conducted to evaluate polarization. The results showed that EVs reduced CD86 levels in M1 cells, and increased CD206 levels in M2 cells (supplementary Fig. S4a,b). The results demonstrate that EVs inhibit M1 polarization and promote M2 polarization. Taken together, EVs released from CAFs facilitate macrophage polarization from M1 to M2 type by upregulating Dicer expression, suggesting that CAF-EVs carried miRNAs to serve their functions.

### miR-4253 expression is elevated in CAF-EVs

**Figure 4.**
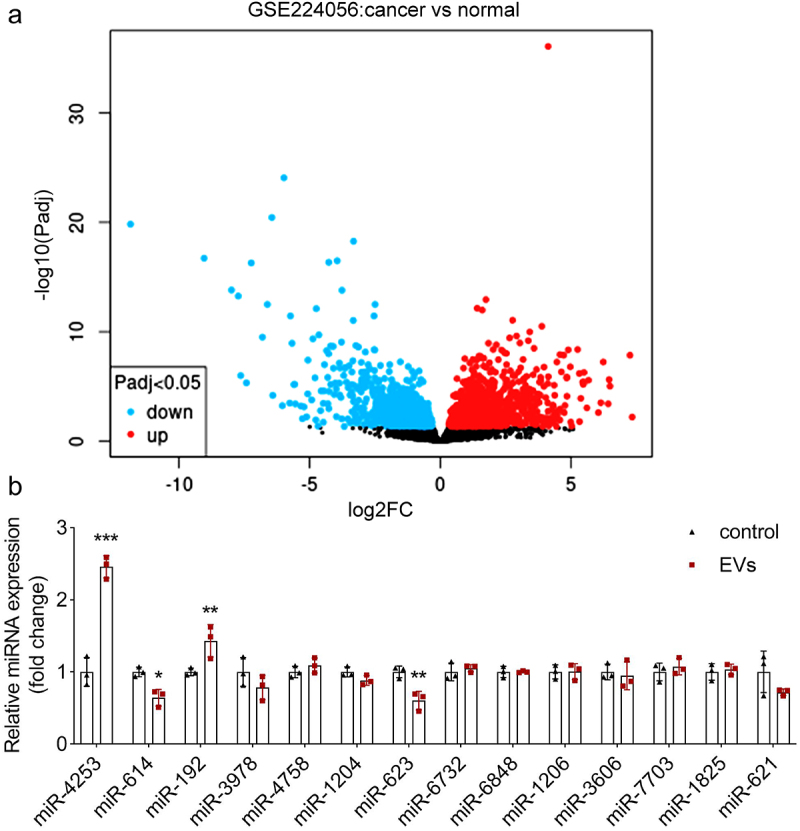
miR-4253 expression is elevated in CAF-EVs. (a) Differentially expressed miRNAs in GC were predicted using GSE224056 microarray analysis and shown using the volcano plot. Blue: downregulated genes. Red: upregulated genes. (b) The expression of all upregulated miRNAs predicted by microarray analysis was measured using qPCR. ***p < .001 and **p < .01. EV, extracellular vesicle.

To investigate which miRNA is involved in macrophage polarization, we used microarray analysis to predict differentially expressed miRNAs in GC. The volcano plot showed numerous upregulated and downregulated miRNAs in GC, compared with normal (Figure 4a). As mentioned above, CAF-EVs promote M2 polarization, which may accelerate GC progression. Therefore, we chose upregulated miRNAs that may have tumor-promoting effects. The results showed that miR-4253 and miR-192 were highly expressed, and miR-623 expression was downregulated in CAF-EVs. The other miRNAs had no significant difference between the two groups (Figure 4b). The miR-4253 with the highest expression was chosen for the following study.

### EV-packaged miR-4253 facilitates M1 to M2 macrophage polarization

**Figure 5.**
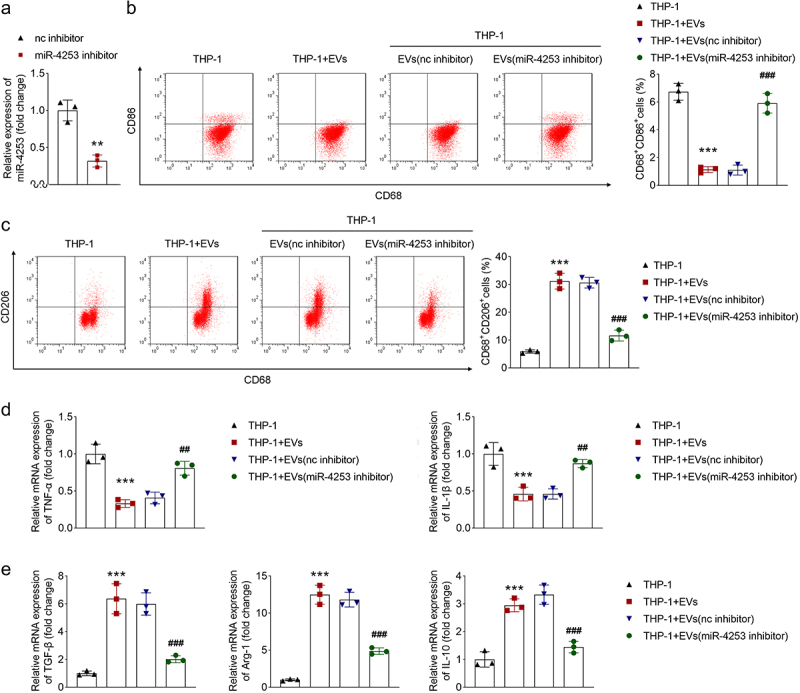
Ev-encapsulated miR-4253 facilitates M1 to M2 macrophage polarization. (a) qPCR was used to determine miR-4253 expression following the miR-4253 inhibitor and nc inhibitor transfection. (b, c) Flow cytometry was conducted the measure CD86 and CD206 expression, i.e. CD68^+^CD86^+^ and CD68^+^CD206^+^ cells, respectively. (d) M1 markers (tnf-α and IL-1β) and (e) M2 markers (tgf-β, Arg-1, and IL-10) expression was examined using qPCR. ***p < .001 and **p < .01 vs. The nc inhibitor or THP-1 group. ###*P* < .001 and ##*P* < .01 vs. The THP-1 + EVs (nc inhibitor) group. EV, extracellular vesicle; nc, negative control.

To analyze the impact of miR-4253 on macrophage polarization, miR-4253 inhibitor and nc inhibitor were transfected into CAFs, and the expression of miR-4253 was downregulated following miR-4253 inhibitor transfection (Figure 5a). Subsequently, EVs were isolated from the transfected CAFs and were used to treat macrophages. The results of flow cytometry showed that EVs inhibited CD86 levels and upregulated CD206 levels, while downregulation of miR-4253 abrogated the effects induced by EVs (Figure 5b,c). qPCR indicated that M1 markers (TNF-α and IL-1β) expression was decreased, and M2 markers (TGF-β, Arg-1, and IL-10) expression was elevated by EV treatment, whereas miR-4253 inhibitor counteracted their levels mediated by EVs (Figure 5d,e). Collectively, EV-packaged miR-4253 inhibits M1 polarization and promotes M2 polarization of macrophages.

### IL6R is a target of miR-4253

**Figure 6.**
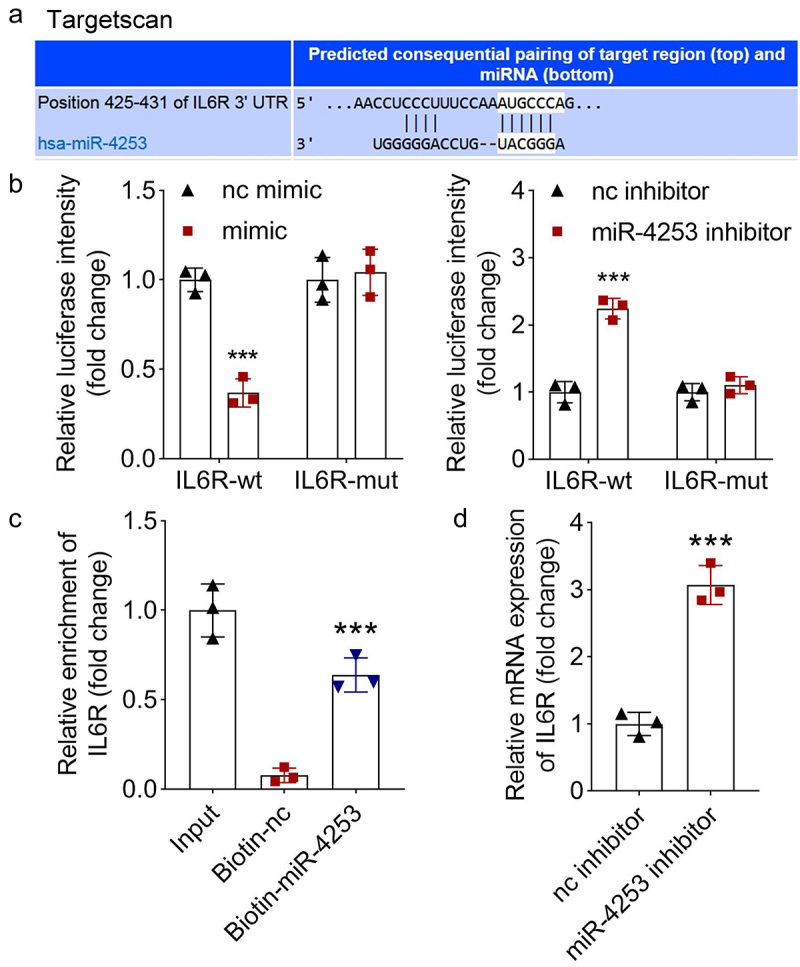
IL6R is a target of miR-4253. (a) Prediction results of miR-4253 targeting IL6R. (b) Dual-luciferase reporter assay and (c) RNA pull-down were carried out to assess the relation of miR-4253 and IL6R. (d) IL6R expression was measured using qPCR after downregulating miR-4253. ***p < .001. nc, negative control.

To investigate the molecular mechanism, we predicted the targets of miR-4253. IL6R might be a target because it had binding sites at 3’UTR to miR-4253 (Figure 6a). Subsequently, the targeting relationship between miR-4253 and IL6R was verified in THP-1 cells. Overexpression of miR-4253 reduced the luciferase activity when co-transfected with IL6R-wt, and downregulation of miR-4253 enhanced that of IL6R-mut (Figure 6b), confirming their targeting relation. RNA pull-down assay verified the interaction between miR-4253 and IL6R (Figure 6c). Moreover, miR-4253 inhibitor elevated IL6R expression in THP-1 cells (Figure 6d). In summary, IL6R is a miR-4253 target.

### EV-packaged miR-4253 regulates macrophage polarization by targeting IL6R

**Figure 7.**
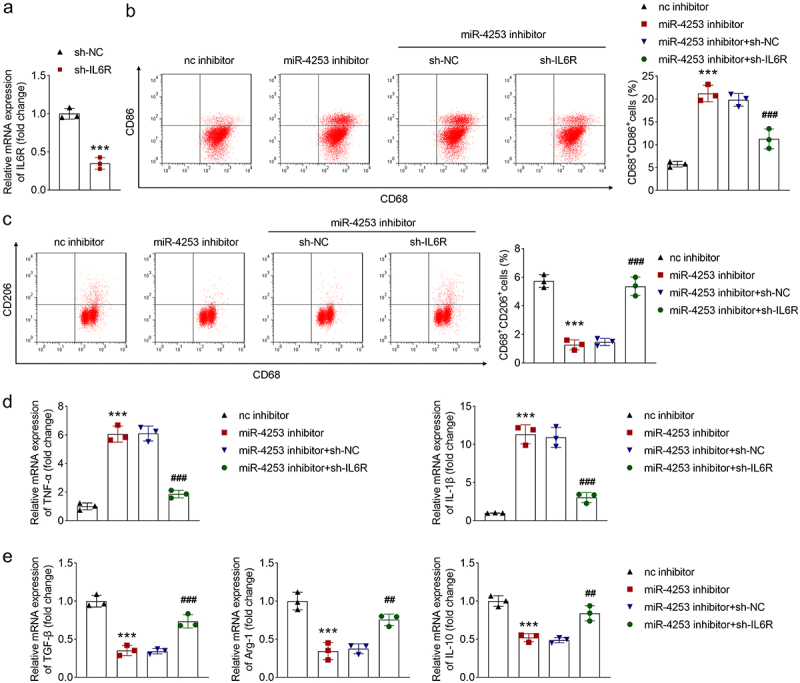
Ev-packaged miR-4253 regulates macrophage polarization by targeting IL6R. (a) IL6R expression was measured in sh-nc and sh-IL6R transfected cells. (b) CD68^+^CD86^+^ and (c) CD68^+^CD206^+^ cells were detected using flow cytometry. (d) TNF-α, IL-1β, (E) tgf-β, Arg-1, and IL-10 expression was examined using qPCR. ***p < .001 vs. The sh-nc or nc inhibitor group. ###*P* < .001 and ##*P* < .01 vs. The miR-4253 inhibitor + sh-nc group. nc, negative control; sh, short hairpin RNA.

To evaluate whether IL6R regulates macrophage polarization, its expression was knocked down in THP-1 cells. After sh-IL6R transfection, its expression was decreased (Figure 7a). Moreover, IL6R knockdown reversed the increase of M1 markers (CD86, TNF-α and IL-1β) and the reduction of M2 markers (CD206, TGF-β, Arg-1, and IL-10) caused by miR-4253 inhibitor (Figure 7b-e). Together, miR-4253 targets IL6R to promote macrophage M2 polarization from the M1 type.

### THP-1 cells treated with EVs carrying miR-4253 promote GC cell proliferation

**Figure 8.**
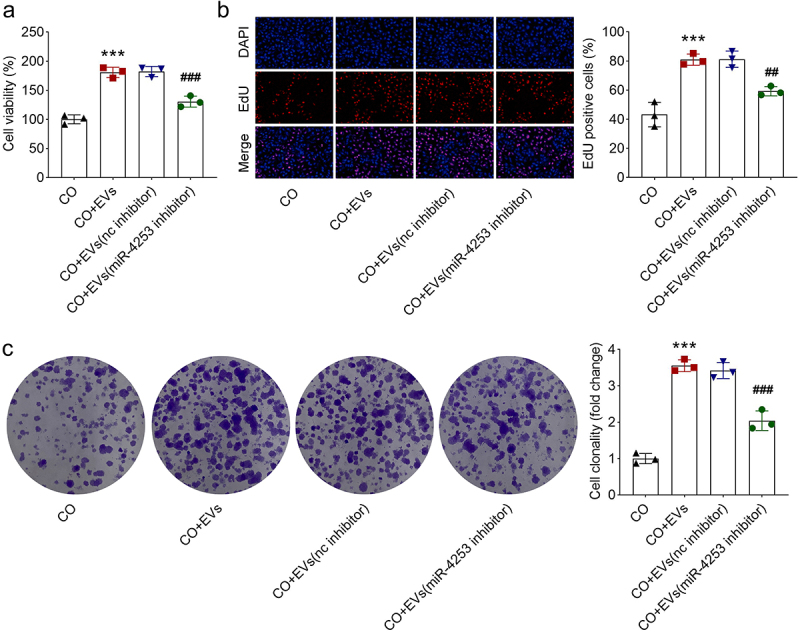
THP-1 cells treated with EVs carrying miR-4253 promote GC cell proliferation. GC cell proliferation was analyzed by (a) CCK-8, (b) EdU assay, and (c) colony formation assay. ***p < .001 vs. The co-culture (CO) group. ###*P* < .001 and ##*P* < .01 vs. The CO + EVs (nc inhibitor) group. CO, co-culture; EV, extracellular vesicle; nc, negative control.

AGS cells were co-cultured with EV-treated macrophages, and the proliferation of AGS cells was determined using CCK-8, EdU assay, and colony formation assay. As shown in Figure 8a-c, THP-1 cells treated with CAF EVs promoted GC cell proliferation. In addition, miR-4253 inhibitor was added to downregulate miR-4253 expression, and the results showed that miR-4253 inhibitor partly abrogated the promotion of GC cell proliferation (Figure 8a-c). The results demonstrate that EVs carrying miR-4253 facilitate the proliferation of GC cells, at least in part by increasing M1 to M2 macrophage polarization.

## Discussion

CAFs play a critical role in cancer progression by releasing EVs, which act as carriers of multiple signals, such as nucleic acids, lipids, and proteins. For example, CAF-released EVs promote cellular proliferation, migration, invasion, and tumor growth in colorectal cancer by regulating the miR-345-5p/CDKN1A axis.^21^ Additionally, CAF-derived miR-146a-5p through EVs facilitate cancer stemness and resistance to gemcitabine or cisplatin.^22^ Notably, CAFs communicate with macrophages in TME by secreting EVs. Macrophage polarization is a key factor in cancer progression. Due to the pro-inflammatory and anti-inflammatory properties of M1-like and M2-like macrophages, they play anti-tumor and pro-tumor roles in malignancy, respectively. It has been reported that CAFs promote the polarization of macrophages to the M2 phenotype in several types of cancers, such as breast cancer^23^ and hepatocellular carcinoma.^24^ Notably, Qiu et al.^25^ have reported that M2-like macrophage promotes angiogenesis, and then facilitates GC metastatic to liver. Li et al.^26^ have found that M2 macrophage polarization caused by GC-derived mesenchymal stromal cells contributes to accelerating cellular migration, invasion, and EMT. A previous study has revealed that IGFBP7 derived by CAFs enhances M2 polarization, suggesting the important effect of CAFs on macrophage polarization in GC.^27^ However, little is known about the implication of CAF-released EVs in macrophage polarization in GC. THP-1 is a human leukemia monocytic cell line. It can differentiate into a state similar to macrophages under specific conditions, and is often used as a substitute for human primary macrophages. It has been widely used to study the function and mechanism of monocytes/macrophages in vitro.^28,29^ Several previous studies used the THP-1 cells to explore the effect of CAFs on macrophage polarization in cancers.^24,30^ In the present study, we treated THP-1 cells with PMA to induce differentiation into macrophages. We found that CAFs promoted M2 polarization and suppressed M1 polarization of macrophages, partly by releasing EVs. Moreover, CAF-derived EVs facilitate GC cell proliferation by modulating macrophage polarization in vitro. The findings suggest that CAF-EVs may accelerate GC progression by inducing macrophage M2 polarization.

EVs have been confirmed to package miRNAs, which are released into target cells to regulate gene expression post-transcription, thereby modulating cellular processes.^31,32^ EV-packaged miRNAs in malignancy can affect several biological functions, such as tumor growth, metastasis, and drug resistance.^33^ For instance, CAF-released EVs facilitate the malignant properties of GC cells via carrying miR-199a-5p.^34^ Besides, CAF-secreted EV-packaged miR-29b-1-5p targets VSIG1 to promote cellular invasive and migrative capabilities to expedite GC progression.^35^ Herein, we predicted that miR-4253 was highly expressed in GC, and confirmed its high expression in CAF-EVs. Increased miR-4253 expression is linked to the death risk of esophageal adenocarcinoma.^36^ Nevertheless, the role of miR-4253 in GC is unknown. In the present study, the results demonstrated that downregulation of miR-4253 counteracted the inhibition of M1 polarization and the promotion of M2 polarization caused by CAF-derived EVs. Moreover, the miR-4253 inhibitor abrogated cell proliferation that was promoted by EV-treated macrophages. The findings suggest that EV-encapsulated miR-4253 functions as an oncogene in GC.

IL-6 is an inflammatory factor that exhibits both pro-inflammatory and anti-inflammatory functions.^37^ During the initial stage of inflammation, upregulation of IL-6 promotes inflammation, whereas a sustained inflammatory response inhibits IL-6 signal transduction to control inflammation. IL-6 regulates several cellular functions by acting on its receptor IL6R or gp130.^38^ Previous studies have indicated that IL6R is involved in macrophage polarization. Weng et al.^39^ have found that MCT-1 promotes the enrichment of M2-like macrophages in TME by stimulating IL-6 release and increasing IL6R levels in breast cancer. Additionally, Miao et al.^40^ have revealed that STING regulates TAM polarization to pro-inflammatory phenotypes by activating the IL6R in GC. Due to the close relationship between IL-6 R and macrophages, and IL6R was identified as a target of miR-4253, we analyzed the effect of IL6R on macrophage polarization in GC. We found that knockdown of IL6R reversed the M2/M1 macrophage polarization induced by downregulating miR-4253. The findings suggest that EV-packaged miR-4253 induces M2-like macrophage polarization to promote GC progression by targeting IL6R.

There are still some limitations in this study. We only used THP-1 cells as the macrophages, which is fundamentally different from TAMs. Primary macrophages from peripheral blood samples are needed to support our conclusion. Additionally, CAFs used in our study were purchased. Although they are also primary cells, we do not know the basic information about these patients. It is not known whether CAFs from different patients with GC have different effects on macrophage polarization. In our future work, we will collect CAFs from several patients with GC. We will analyze the basic clinical characteristics, and investigated the effects and mechanisms of CAFs from patients of different types and stages on macrophage polarization and tumor cell growth. We consider that addressing these limitations will help us more clearly elucidate the communication between the two cells and further support our conclusions.

In conclusion, EV-packaged miR-4253 from CAFs promotes M1 to M2 macrophage polarization via targeting IL6R, thereby facilitating the proliferation of GC cells, which may accelerate GC progression. The findings suggest that targeting EV-packaged miR-4253 may be a promising therapeutic strategy for GC.

## Supplementary Material

Supplemental Material

supplementary Fig 1.jpg

supplementary Fig 3.jpg

supplementary Fig 4.jpg

supplementary Fig 2.jpg

## Data Availability

The datasets used and/or analyzed during the current study are available from the corresponding author on reasonable request.
