# Supplementary material for: Extracellular vesicle-packaged miR-4253 secreted by cancer-associated fibroblasts facilitates cell proliferation in gastric cancer by inducing macrophage M2 polarization
Source: Cancer Biol Ther. 2024 Nov 6;25(1):2424490. doi: 10.1080/15384047.2024.2424490 (PMC11542604; doi:10.1080/15384047.2024.2424490)
Supplement: Supplemental Material [file KCBT_A_2424490_SM0326.docx]

Supplementary Figure 1. Identification of CAFs. (A) Cellular morphology was observed under a light microscope. (B) Immunofluorescence was performed to determine the expression of αSMA and FAP. 4′,6-diamidino-2-phenylindole (DAPI) was used to stain nuclei. CAF. cancer-associated fibroblast.

Supplementary Figure 2. GW4869 reduces the number of EVs. EVs were isolated from CAFs treated with GW4869 or not, and THP-1 cells were incubated with EVs. The levels of EV markers CD63 and TSG101 were measured using immunoblotting. CAF. cancer-associated fibroblast; EV, extracellular vesicle.

Supplementary Figure 3. Effect of GW4869 on macrophage polarization. THP-1 cells were treated with GW4869 alone or not, and (A) CD68^+^CD86^+^ and (B) CD68^+^CD206^+^ cells were measured using flow cytometry.

Supplementary Figure 4. Effect of EVs on polarization of M1 and M2 cells. THP-1 cells were induced into M1 or M2 macrophages and then treated with EVs. Macrophage polarization was assessed by detecting (A) CD68^+^CD86^+^ and (B) CD68^+^CD206^+^ cells using flow cytometry. ***P < 0.001. EV, extracellular vesicle.
