# Supplementary figures and images for: Extracellular vesicle-packaged miR-4253 secreted by cancer-associated fibroblasts facilitates cell proliferation in gastric cancer by inducing macrophage M2 polarization
Source: Cancer Biol Ther. 2024 Nov 6;25(1):2424490. doi: 10.1080/15384047.2024.2424490 (PMC11542604; doi:10.1080/15384047.2024.2424490)

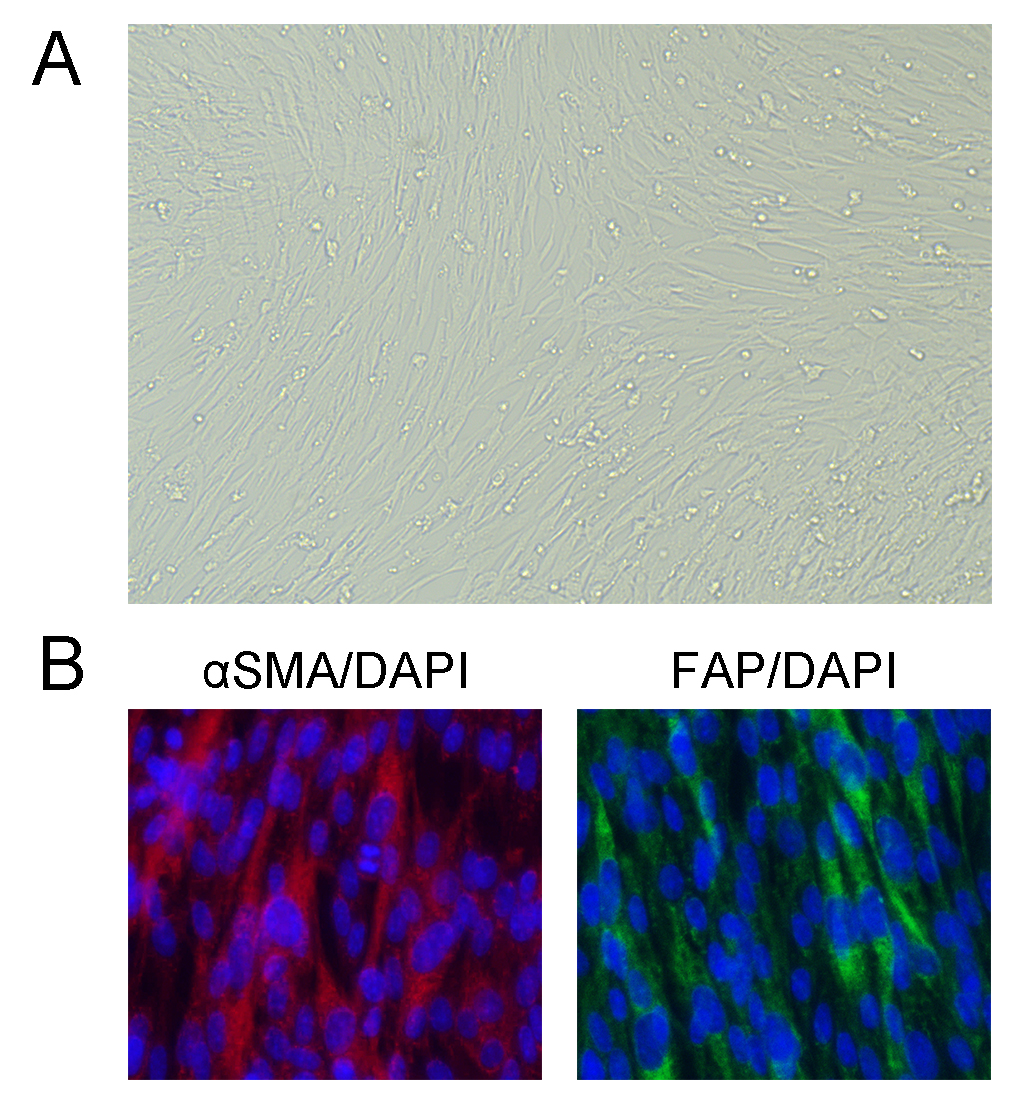

Supplement: supplementary Fig 1.jpg [file KCBT_A_2424490_SM3439.jpg]

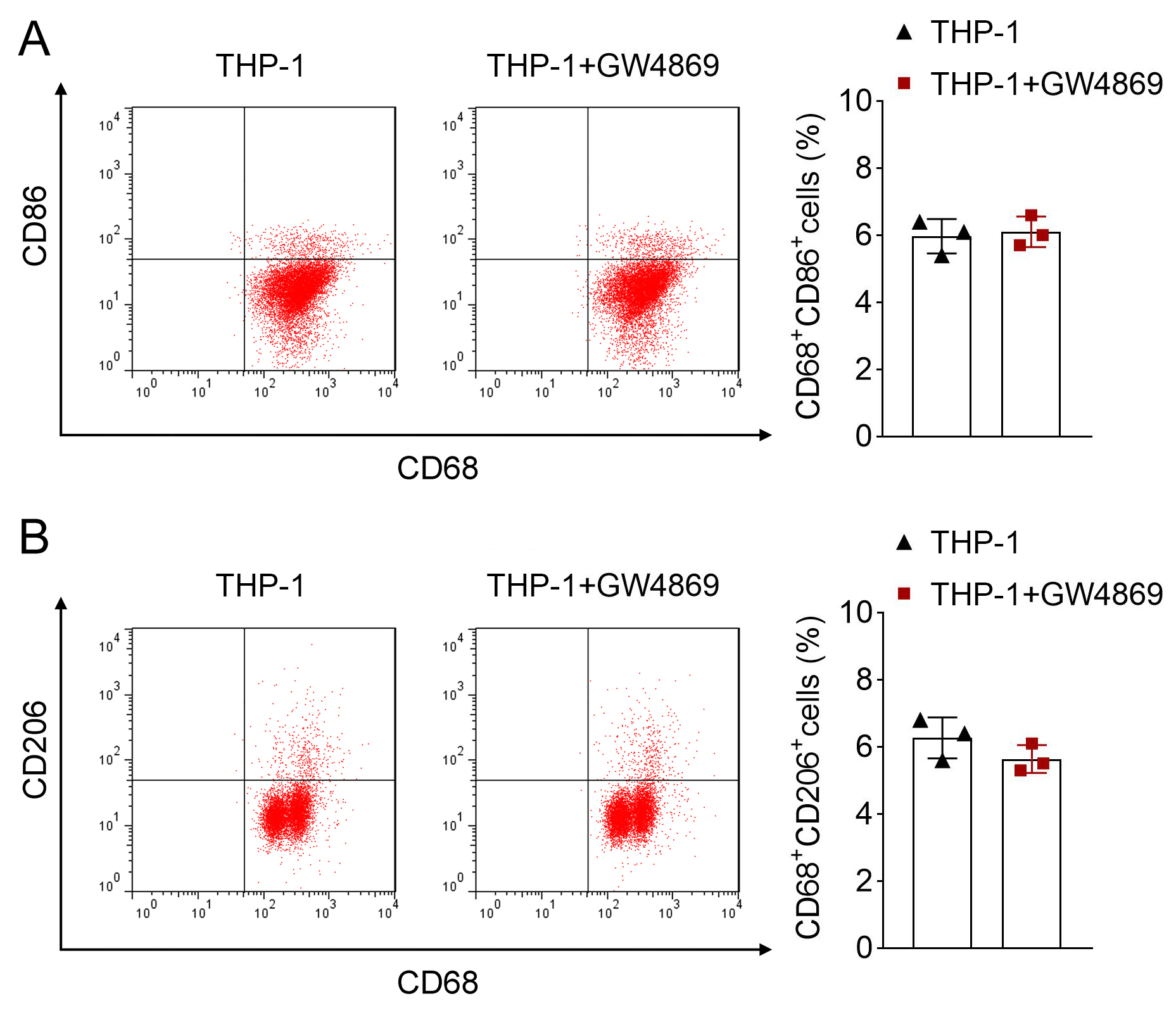

Supplement: supplementary Fig 3.jpg [file KCBT_A_2424490_SM3438.jpg]

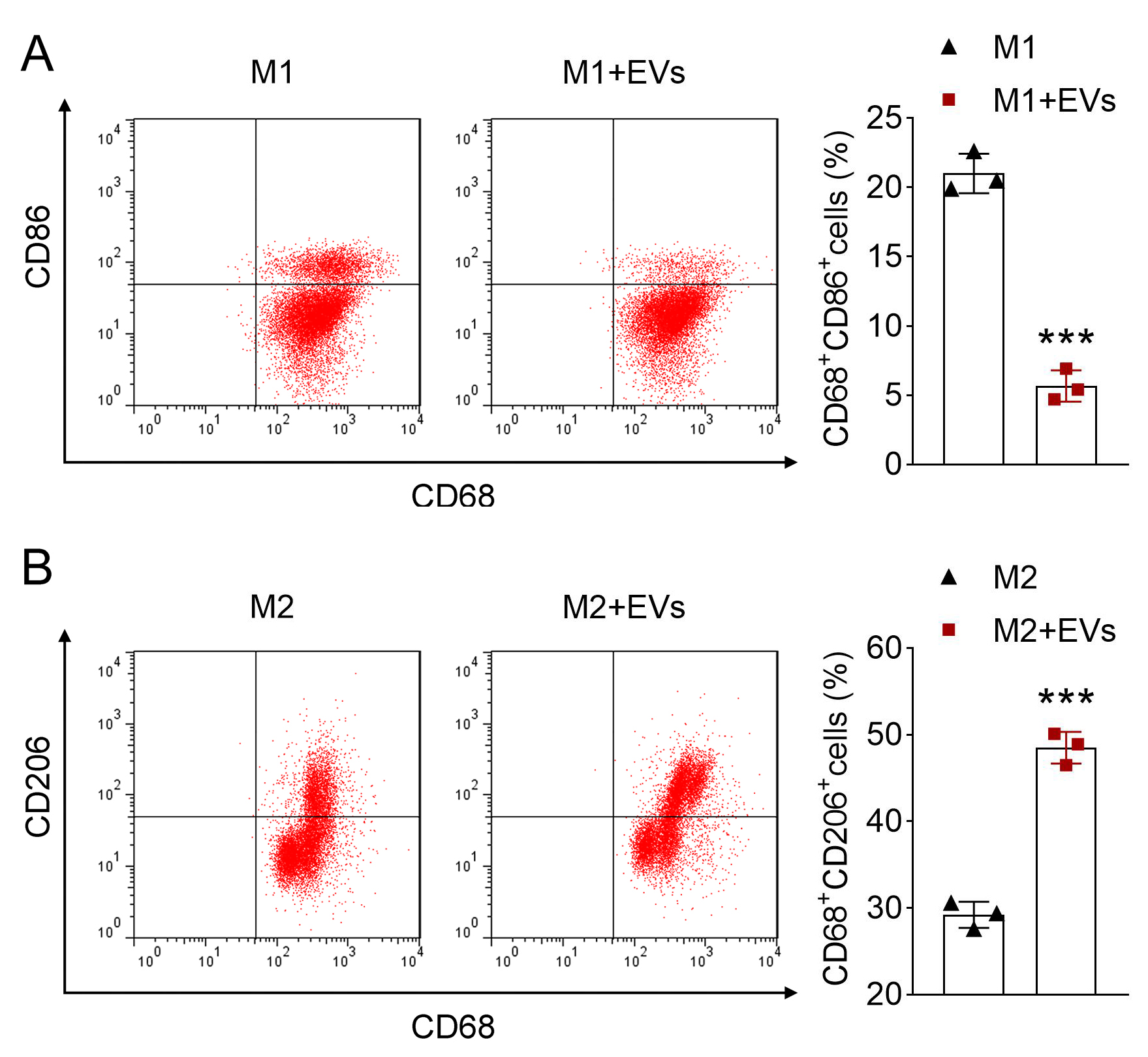

Supplement: supplementary Fig 4.jpg [file KCBT_A_2424490_SM3437.jpg]

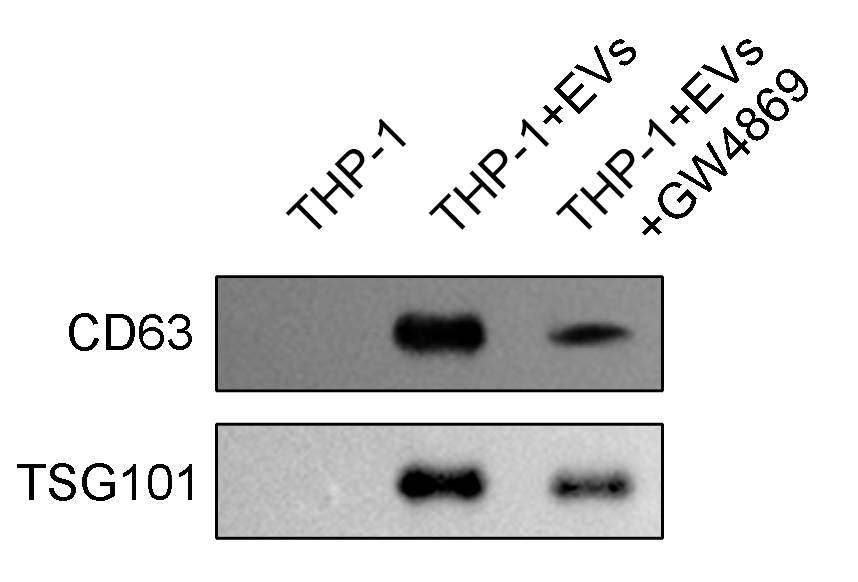

Supplement: supplementary Fig 2.jpg [file KCBT_A_2424490_SM3436.jpg]
